# Toward transferable models for efficient spatiotemporal flood prediction across coastal-estuarine systems

**DOI:** 10.1017/cft.2026.10037

**Published:** 2026-06-04

**Authors:** Samuel Daramola, David F. Muñoz, Chaopeng Shen

**Affiliations:** 1 Department of Civil, Environment, and Ocean Engineering, Stevens Institute of Technology, NJ, USA; 2 Department of Civil and Environmental Engineering, Virginia Tech, Blacksburg, VA, USA; 3 Department of Civil and Environmental Engineering, Pennsylvania State University, PA, USA

**Keywords:** transfer learning, spatiotemporal flood prediction, physics-informed machine learning, coastal-estuarine hydrodynamics, extreme water levels

## Abstract

This perspective examines the application of transfer learning (TL) within deep learning (DL) frameworks for extreme water level (EWL) and spatiotemporal flood predictions. We discuss the main advantages of TL, such as enabling model transferability of pretrained DL models from/to diverse coastal-estuarine systems and reducing computational time compared to physics-based models. These advantages can accelerate the deployment of flood prediction models in data-limited locations. We also discuss challenges and limitations that hinder accurate pattern recognition and propagation of EWLs from gauge (observation) stations to surrounding locations within model domains. These limitations include dependence on similarity in data distributions, overfitting the training data and both lag and hysteresis effects in the timing of peak water levels and flood dynamics. Lastly, we explain several misconceptions and challenges in current DL approaches that hinder EWL and spatiotemporal flood prediction, including training models exclusively on extreme conditions, assessing accuracy solely through goodness-of-fit metrics, and connecting the model’s knowledge of input data with physical explanations of flood processes without an adequate context. We argue that these challenges can be addressed by prioritizing storm-relevant patterns of the input data features as well as embedding spatial propagation of EWLs in DL frameworks to mimic coastal-estuarine hydrodynamic models. Ultimately, progress toward generalizable model transferability relies on the modeler’s ability to incorporate physical understanding in DL architectures, alongside continued advances in physics-informed machine learning models via soft or hard constraint approaches. There remains substantial work to establish guidelines and/or formal procedures to develop robust, interpretable, and generalizable DL models for spatiotemporal flood prediction, thereby supporting effective flood management, mitigation, and emergency preparedness.

## Graphical abstract

**Figure:**
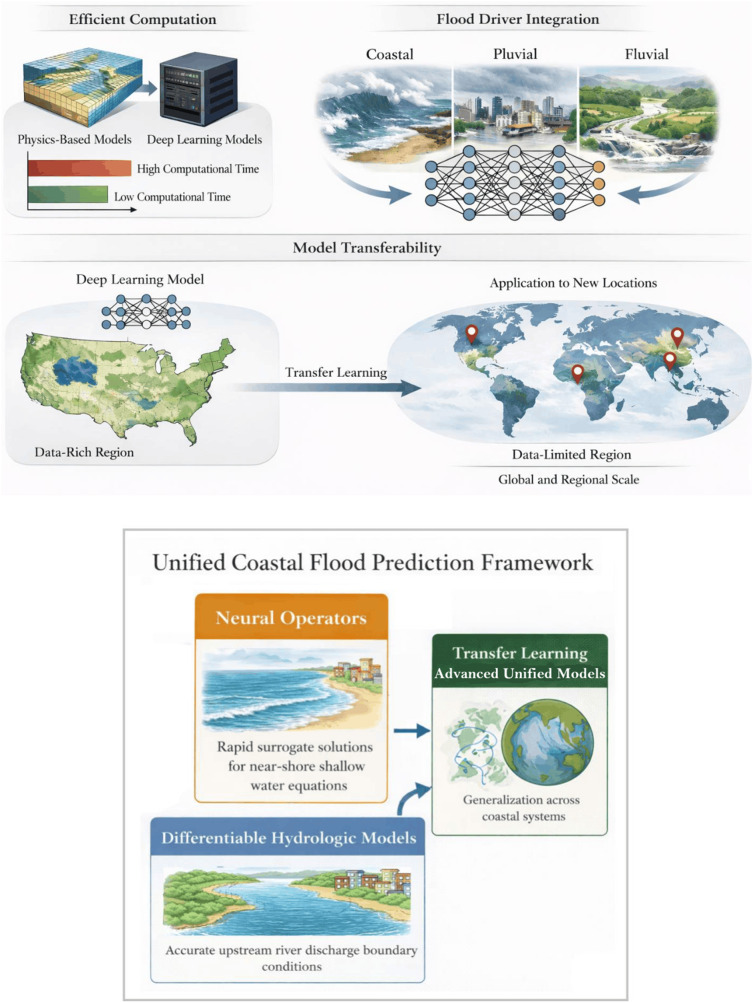

## Impact statement

As flood risks intensify across coastal and estuarine regions, communities need widely deployable prediction systems that are not only accurate but also fast, scalable and usable in places with limited data. This perspective shows how deep learning and transfer learning can help meet that need by reducing computational demands, integrating multiple flood drivers within a unified framework and extending predictive capability from data-rich regions across coastal-estuarine systems. It advances the field by reframing flood machine learning from a site-specific prediction problem into a transferability problem. In this way, it points toward a future in which flood forecasting becomes more accessible and practical for emergency planning, hazard mitigation and infrastructure resilience at the local, regional and global scales. Beyond identifying opportunities, this perspective explains why current models often fail when transferred from one coastal system to another. It shows that transferable flood prediction requires more than statistical accuracy: models must capture physically meaningful patterns, represent the timing and propagation of extreme water levels realistically and remain robust when environmental conditions differ across regions. By arguing for physics-informed machine learning that emphasizes storm-relevant signals and spatial propagation consistent with coastal-estuarine dynamics, this article offers a practical roadmap for building models that are not only faster but also more interpretable and trustworthy in unfamiliar settings. The broader impact is a shift in the field from site-specific flood prediction toward transferable, next-generation forecasting systems, laying the foundation for large pretrained hydrologic and hydrodynamic models that can accumulate knowledge across many environments and expand equitable access to advanced flood prediction technology worldwide.

## Advancing flood prediction with deep learning and transfer learning techniques

Flood prediction at point-based tide gauges and/or across spatial domains has been significantly improved through the application of deep learning (DL) models (Shen, 2018; Bentivoglio et al., 2022). Given adequate training data, DL models in the water resources field achieve predictive accuracies comparable to physics-based models while requiring substantially lower computational time (Shen et al., 2023; Kratzert et al., 2024a, 2024b). DL models with specialized architectures can effectively capture nonlinear interactions among coastal, pluvial and fluvial flood drivers and processes, positioning them as critical tools for bolstering resilience against escalating flood risks (Oddo et al., 2024; Liu et al., 2025). Yet, efficient deployment and execution of regional flood prediction models remain urgently needed to support decision-making and emergency preparedness. Physics-based (or process-based) models that solve the Navier–Stokes or simplified shallow-water equations deliver accurate flood predictions under appropriate input and/or forcing conditions, but they are site-specific, which renders them impractical for widespread use in data-limited and/or data-scarce locations (Santiago-Collazo et al., 2019; Bates et al., 2021; Bates, 2023). To avoid training site-specific models, transfer learning (TL) within DL frameworks leverages pretrained models in data-rich locations for immediate application to unseen locations (Chen et al., 2021; Immorlano et al., 2025), and in some cases via zero-shot applications, where a pretrained model is applied to a target location without site-specific fine-tuning (Daramola et al., 2025b). Nevertheless, TL has not been fully developed for accurate spatiotemporal flood prediction nor tested on a continental and/or global scale.

## Challenges of spatiotemporal transfer learning

**Figure 1.**
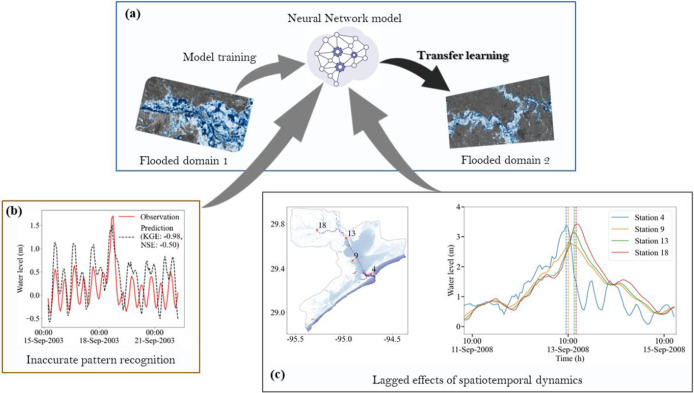
Schematic illustration of the neural network-based transfer learning framework for flood modeling and challenges. (a) The model is first trained on flooded domain 1 and then transferred to a different target domain (flooded domain 2). The lower panels highlight two main limitations: (b) inaccurate pattern recognition at a tide gauge, where predictions fail to reproduce observed water levels (WLs), and (c) lagged effects of spatiotemporal dynamics, where peak WLs occur at different times across stations in the target domain. Figure 1. long description.

The main challenges of DL frameworks for spatiotemporal flood prediction are inaccurate pattern recognition and lag and hysteresis effects of spatiotemporal dynamics (Figure 1). Inaccurate pattern recognition primarily reduces TL accuracy, so similarity between source and target data distributions is often required.

### Similarity in data distributions, overfitting and hyperparameter configurations

TL’s success depends critically on sufficient similarity between the source and target domains, particularly in the data distributions of predictor features, such as similar frequencies and magnitudes of anomalies (Lee et al., 2021; Hamitouche and Molina, 2022). When this requirement does not hold, dataset shift occurs, whereby differences in data distributions undermine model generalization and can lead to negative transfer (Moreno-Torres et al., 2012; Chen et al., 2021; Xu et al., 2023). Such dataset shifts may also emerge over time within the same coastal-estuarine system, as sea level rise, changing baseline water levels, subsidence or anthropogenic modifications alter the distribution of WLs and flood drivers (Ohenhen et al., 2026). Overfitting also limits effective TL, where DL models learn overly specific details from the training data and fail to capture generalizable patterns between source and target domains (Pan and Yang, 2010; Peng et al., 2022). Overfitting introduces noise into the transfer process and undermines predictive performance in the target domain, especially when training datasets are limited to extreme conditions or are unrepresentative of broader flooding scenarios. Moreover, models are typically developed by tuning hyperparameters to the specific conditions of the training domain, to maximize accuracy on the training set. The strong dependence of optimal hyperparameters on particular datasets complicates the effectiveness of TL, as these configurations may not transfer well to the target domain (Ma et al., 2020; Tran et al., 2020; Dong et al., 2021; Marco et al., 2022; Wang et al., 2023).

### Lagged and hysteresis effects

Conventional DL models often lack an explicit representation of how extreme water levels (EWLs) and associated flooding propagate from a point source across space and do not inherently encode storm-track direction (Lee et al., 2021; Zhang et al., 2025). As a result, they struggle to capture spatially varying lags at the timing of peak water levels (WLs). For example, peak WLs recorded at coastal locations can occur hours to days before those of upstream or inland areas, and importantly, such lags vary nonlinearly in space and time (Xiao et al., 2021; Dykstra et al., 2022). Spatially, WL variations at one location influence adjacent areas through physical forcing mechanisms such as bottom friction that controls flood wave propagation and dissipation of energy in coastal-estuarine systems. Likewise, coastal hydrodynamic nonlinear interactions (Xiao et al., 2021; Moftakhari et al., 2024; Sakib et al., 2025), together with morphology, can either amplify or attenuate WLs (Prandle, 1985; Hoitink and Jay, 2016; Talke and Jay, 2020). Temporally, the timing of peak WLs varies across inland, transition and coastal regions due to factors like distance from the flood source, bathymetry, backwater effects and flood connectivity, introducing lagged and hysteresis effects (Brunner et al., 2020; Talke and Jay, 2020). If models implicitly assume near-simultaneous peak occurrences across a spatial domain, they can yield erroneous lead or lag time predictions, resulting in overestimation or underestimation of flood impacts (Yu et al., 2024). When flood propagation from point sources is not adequately represented in the DL architecture, transferred models tend to misalign the magnitude and timing of flood peaks across space.

## Misconceptions of deep learning modeling

Common misconceptions in DL modeling can contribute to challenges in spatiotemporal flood prediction, particularly in how models are developed, evaluated and interpreted.

### Training DL models only on extreme conditions to improve the model’s performance

Training DL models only on extreme conditions could be disadvantageous for two main reasons. First, there might be insufficient training data for extreme events (or WLs) at any area (or gauge station). As an illustrative example, and recognizing that extreme event frequency and duration vary substantially by region and threshold definition (e.g., annual maxima, peaks-over-threshold or return periods), events lasting about 2–4 days and occurring twice every 5 years in a given area would amount to only about 16 events, or 32–64 extreme days out of ~14,600 days in over 40 years. Such a limited number of extreme events is typically inadequate for training a DL model unless supplemented with many synthetic events. Second, a model trained exclusively on extreme conditions does not recognize sudden changes or anomalies between normal and extreme conditions, even though distinguishing these regimes is a primary objective of flood prediction models. In that case, it is unclear whether the model has truly learned to identify extreme events. It is only by training on the full historical record while explicitly emphasizing extreme periods (e.g., through loss reweighting or architectural choices) that we can be more confident that the model will accurately detect the timing and occurrence of extremes.

### Assessing accuracy solely on goodness-of-fit metrics

Model accuracy in DL flood prediction is often evaluated based on favorable goodness-of-fit metrics. However, this approach can be deceptive, particularly for imbalanced datasets emerging in flood modeling. For example, in a dataset of 10,000 instances where only 500 represent flood events, a model that predicts “no flood” for every case would still achieve 95% accuracy, even though it fails to identify any flood events. Also, accurate prediction of peak WL magnitude is often prioritized, with timing considered secondary once validation accuracy exceeds a high threshold (Dong et al., 2021). Yet, small timing errors between predicted and actual peaks of storm tide can lead to large discrepancies in flood impact assessments, underscoring the need for precise timing in spatiotemporal analyses. Correct temporal variations are critical for effective real-time flood response, particularly in large domains with heterogeneous flood dynamics.

### Explainable deep learning

**Figure 2.**
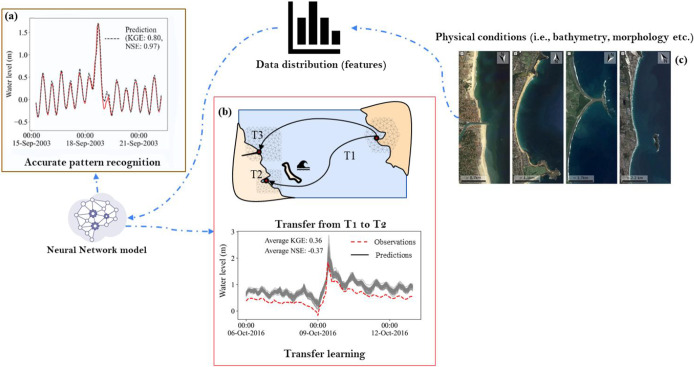
Conceptualization of the influence of data distribution and physical conditions in transfer learning performance for extreme water level (EWL) prediction. The deep learning (DL) model is first trained at an ocean-exposed station (domain 1), where it achieves accurate pattern recognition of EWLs (panel a). When the DL model is transferred to a barrier-protected station (domain 2) of a different morphologic setting, shifts in predictor distributions lead to degraded performance in terms of the average KGE and NSE values (panels b and c). Figure 2. long description.

The ability of DL models to identify complex nonlinear interactions between input features and target variables is sometimes misinterpreted as evidence that they understand the underlying physics. This raises concerns about their interpretability, as tools such as SHAP, LIME and permutation importance primarily quantify the contribution of predictor variables to training accuracy rather than their physical relevance to flood processes. For DL models, these nonlinear interactions are learned from the data distributions (Reichstein et al., 2019), and TL is typically more accurate when this distribution is similar in both training and test sets (Gong et al., 2016; Zhuang et al., 2020), even in the absence of explicit physical understanding. Data distributions are shaped by physical conditions (Figure 2), including topography, hydrological and climatic characteristics, which influence critical variables such as WLs (Marcos et al., 2019; Serafin et al., 2019; Almar et al., 2021; Costa et al., 2023). Physical differences across geographical domains can lead to divergent data distributions, posing a significant challenge for TL techniques (Daramola et al., 2025b, 2026).

## Extreme water level patterns and propagation from observation stations

The challenges of TL across coastal-estuarine systems can be addressed by following two steps:

### Prioritize storm-relevant extreme patterns in the learning of the water level signal

First, the selection of input features and DL architectures should be informed by physical processes that capture the events’ EWLs and associated flood dynamics. DL modelers should then aim to ground both the model structure and improvements in training accuracy in the physical drivers of flooding (Kumar et al., 2025). That is, architectural modifications so that modules, data pathways and fusion operations reflect the real physical decomposition and coupling of processes (e.g., tides, surge, river flow, rainfall-runoff and waves). In coastal-estuarine systems subject to compound flooding, TL-based flood models should also evaluate whether these interacting drivers are comparable between source and target domains. Moreover, methodological advances should emphasize training strategies that improve a model’s sensitivity to physically meaningful flood-related signals (e.g., periods of high-WLs, surge, etc.), rather than optimizing primarily for frequent, repetitive patterns in the training data.

When only 32–64 extreme days are available out of ~14,600 days, as in the illustrative example in section “Training DL models only on extreme conditions to improve the model’s performance,” extremes become underrepresented in the training data, making it difficult for DL models to learn and detect EWLs. In such cases, it is important to modify the architecture to focus on what matters most (Daramola et al., 2025b), which is to prioritize storm-relevant patterns in the learning of WL signals (Figure 2a). For example, an attention mechanism (Vaswani et al., 2017) can be introduced and customized to emphasize the influence of EWL periods (Yang et al., 2024), thereby improving the model’s ability to learn and represent these events (Orozco López et al., 2024). In practice, high attention weights often concentrate on time windows where the dynamics are more complex or harder to approximate, which frequently coincide with periods of EWLs (Daramola et al., 2025b). If such techniques are incorporated into DL architectures, models are better positioned to capture flood events in unseen test datasets after the training stage and predict their magnitudes and timing with higher accuracy (Zhang et al., 2025). While such techniques do not fully replace the role of explicit conservation equations, they enhance the capture of sudden and/or extreme changes in the WL signal and ensure internal consistency in the learned data patterns.

### Embed spatial propagation consistent with coastal-estuarine hydrodynamics around physically aware DL frameworks

**Figure 3.**
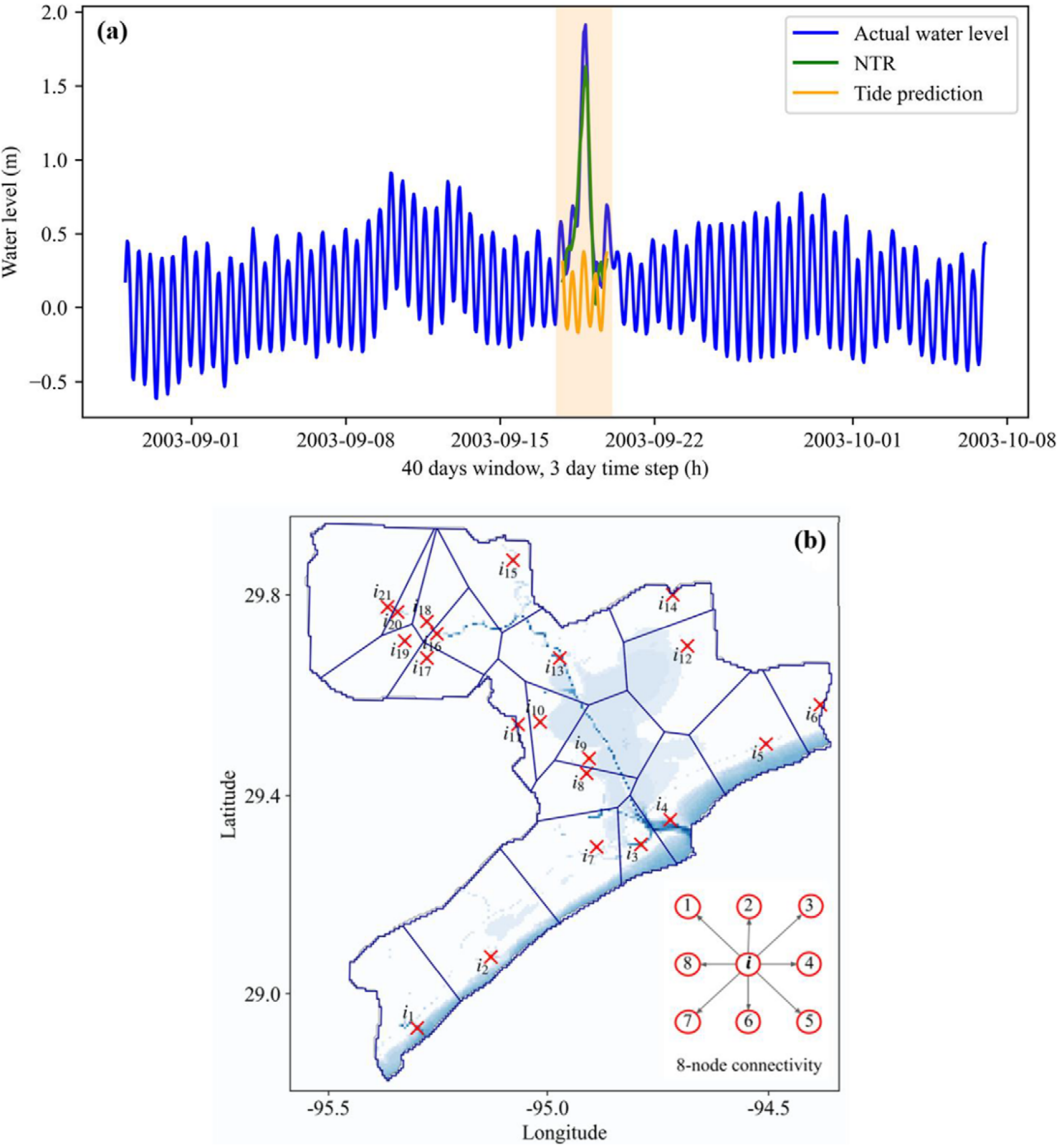
(a) A 40-day time series window of observed water levels, predicted astronomical tides and nontidal residuals (NTR) with the shaded interval highlighting storm-driven extreme period. (b) Study domain showing spatial partitioning (clusters) around tide gauges (red crosses) and the corresponding graph representation. The inset illustrates an eight-node neighborhood connectivity used in the graph convolutional network framework, where each grid cell/node exchanges information with its surrounding neighbors to propagate WL signals from gauge locations across the model domain. Figure 3. long description.

For DL models that aim to propagate EWLs from tide-gauge point sources across a domain, sparse observation networks make it difficult to learn how EWLs evolve spatially (Figure 3b). A relatively simple strategy is to split a model domain into clusters with their centroid coinciding with the location of representative gauges. In that sense, time series from each gauge can represent the coastal-estuarine system’s response of the corresponding cluster to extreme conditions (Daramola et al., 2025a). Nevertheless, this approach does not account for variability within the cluster and thus cannot resolve spatial differences in the timing and magnitude of extremes (Farahmand et al., 2023; Yu et al., 2024; Fathi et al., 2025).

Sophisticated approaches like using encoder-decoder architectures (e.g., U-Net) can transform sparse gauge information into a latent space representation that is then decoded to produce dense WL maps. While this enables propagation from scarce observations to a dense grid, the resulting fields are often closer to a learned interpolation than a true estimation of the underlying dynamics, particularly in regions far from gauges or under unprecedented extreme events. In contrast, graph convolutional networks (GCNs) can be configured with each grid cell treated as a node and edges connecting its neighbors within the model domain (Daramola et al., 2026). Gauge locations act as observed source nodes, and information is propagated iteratively from node to node through the graph until the entire domain is covered. By explicitly using local connectivity to propagate WL signals, GCN-based approaches could provide spatial propagation that is more representative of the expected coastal-estuarine hydrodynamics than cluster-based or purely latent space interpolation approaches; thus, supporting more robust TL across heterogeneous domains. Hence, with the accurate capture of extreme patterns, TL is organized meaningfully rather than on purely statistical probability.

The two steps discussed above are physics-guided machine learning techniques, in which the physical characteristics of extreme events determine feature selection, network architecture and training design, as well as the estimation of model predictions. Hence, physics-guided DL models can explore the connection among physical components, processes and data distributions, which is essential for enhancing the performance and applicability of TL across geographically and morphologically distinctive regions. By elucidating these connections, modelers can develop more informed strategies for selecting appropriate source domains and adapting DL models to account for physical variations. Physical awareness in DL frameworks is, therefore, crucial because it validates whether the chosen input features can improve the model’s performance when compared to physics-based model simulations. On the other hand, it provides information on the similarities and differences in dynamic processes, understanding the conditions that matter for model transferability.

## Directions for practical modeling enhancement

### Establishing physics-informed machine learning

There are three main classes of physics-informed machine learning (PIML), namely, physics-informed neural networks (PINNs), differentiable modeling (DM) and physics-guided machine learning, each with distinct strengths. PINNs enforce the system’s governing equations and associated boundary or initial conditions by embedding them into neural network training, most commonly through residual terms in the loss function (Sun et al., 2022; Qin et al., 2023). By contrast, DM couples process-based equations and learnable components within a fully end-to-end differentiable framework. In DM, the physical equations provide structural priors, whereas neural networks are used to learn parameterizations, unresolved processes or model residuals (Shen et al., 2023). At a broader level, physics-guided ML (section “Extreme water level patterns and propagation from observation stations”) refers to the general strategy of injecting physical knowledge into machine learning models through architectural constraints, regularization, priors, pretraining or other mechanisms to improve physical realism and robustness (Daramola et al., 2026). All three approaches are important for advancing coastal flood prediction, but each has limitations when applied to coastal systems characterized by complex nonlinearity, multiscale spatiotemporal variability and, in some cases, turbulence-affected hydrodynamics.

### Unified coastal-estuarine flood prediction framework

Physics-informed ML approaches can be integrated into a unified flood prediction framework. Here, neural operators serve as rapid surrogate solvers for near-shore shallow water equations, and differentiable hydrological models provide more accurate upstream river discharge boundary conditions. Hence, they enable generalization across diverse coastal systems through TL. For instance, Fourier Neural Operators (FNOs) learn solution operators for parametric partial differential equations (PDEs), mapping initial and boundary conditions directly to solutions without explicit time-stepping and achieving speedups of $10^{2}-10^{5}$ over conventional numerical solvers (Li et al., 2021). Extensions such as Spherical FNOs enable stable long-term rollouts for shallow water dynamics on the sphere (Bonev et al., 2023), while graph-based operator architectures naturally accommodate the unstructured meshes characteristic of coastal domains (Shi et al., 2022).

Sensitivity fidelity also matters. Behroozi et al. (2025) showed that standard neural operators can learn highly accurate solution trajectories while producing substantially inaccurate parameter sensitivities (e.g., $R^{2}>0.99$ for solutions vs. $R^{2}<0.21$ for sensitivities), which could undermine their use in coupled optimization or data assimilation frameworks for coastal applications. Sensitivity-constrained neural operators (SC-FNOs) address this limitation by incorporating gradient supervision, maintaining sensitivity accuracy with only modest additional computational cost (~30–130% increase in training time). Despite these advances, neural operators still face challenges in capturing key near-shore processes, including shock phenomena (e.g., hydraulic jumps and bores), wetting and drying fronts and generalization to out-of-distribution bathymetric conditions.

Importantly, the quality of boundary conditions in coastal–estuarine models depends critically on the accuracy of upstream hydrologic predictions. Recent advances in differentiable hydrologic modeling provide substantially more accurate estimates of river discharge and freshwater inputs to coastal regions than traditional global water models. For example, Ji et al. (2025) introduced a high-resolution, physics-embedded differentiable model that identified previously unrecognized declining trends (>1.5% per year) in freshwater inputs to European estuaries, achieving significantly higher trend prediction accuracy ( $R^{2}=0.68$ ) than established global water models ( $R^{2}<0.46$ ). These models enforce strict mass balance and provide physically interpretable diagnostic variables (e.g., evapotranspiration, baseflow and soil moisture), thereby supporting both physical interpretation and stakeholder communication. Moreover, Song et al. (2026) demonstrated that differentiable hydrologic models outperform LSTM-based models under unseen extreme conditions (return periods >50 years), reducing peak flow errors in 80% of cases. This reflects mass conservation and storage-dependent flow constraints, which enable physically consistent responses to unprecedented inputs. Together, these advances suggest that improved predictions of extreme-event river discharge can provide more reliable boundary conditions for coastal flood models.

TL can leverage accumulated knowledge across systems to learn internal representations of recurring coastal–estuarine and riverine dynamics. For example, Ma et al. (2021) demonstrated that LSTM models pretrained on 671 US basins can be successfully transferred to basins in Chile, Great Britain and China, achieving significantly improved performance even with only 1 year of local data. Moreover, the benefits of TL increase with the size and diversity of the source dataset, confirming that hydrologic systems worldwide share exploitable commonalities. This success, together with the demonstrated scaling benefits of large-data training in differentiable models (Ji et al., 2025), motivates developing advanced unified models that encode general hydrologic and hydrodynamic knowledge (Shen et al., 2023). However, such models should be viewed as a research direction requiring careful global data curation, physics-aware pretraining, out-of-distribution evaluation and safeguards against transferring biases from data-rich to data-sparse regions. In operational frameworks, TL-based flood models must communicate uncertainty clearly, through probabilistic outputs with confidence intervals, to support emergency managers and decision-makers, particularly in data-limited regions.

## Data Availability

Data availability is not applicable to this article as no new data were created or analyzed in this study.

## Long descriptions

### Figure 1. Long description

The figure is divided into three panels labeled a, b, and c.

Panel a, located at the top, shows a flowchart for a Neural Network model. On the left, a satellite image of Flooded domain 1 has a grey arrow pointing to a central brain icon representing the model training phase. From the brain icon, a black arrow labeled Transfer learning points to a satellite image of Flooded domain 2 on the right. Two large grey arrows point from the brain icon down toward panels b and c.

Panel b, at the bottom left, is a line graph titled Inaccurate pattern recognition. The y-axis is Water level in meters, ranging from negative 0.5 to 1.5. The x-axis shows dates from 15-Sep-2003 to 21-Sep-2003. A solid red line for Observation shows lower peaks than a dashed black line for Prediction. The legend notes K G E is negative 0.98 and N S E is negative 0.50.

Panel c, at the bottom right, is titled Lagged effects of spatiotemporal dynamics. It contains a map and a line graph. The map on the left shows a coastal region with stations marked 4, 9, 13, and 18 along a river path. The line graph on the right plots Water level in meters from 0 to 4 against Time in hours from 11-Sep-2008 to 15-Sep-2008. Four colored lines represent the stations. Vertical dashed lines indicate that the peak water levels for Station 4, Station 9, Station 13, and Station 18 occur at different times, demonstrating a temporal lag.

Navigate back to Figure 1..

### Figure 2. Long description

The diagram consists of three main panels and two central icons connected by dashed blue arrows.

Panel a, at the top left, is a line graph titled Accurate pattern recognition. The y-axis is Water level in meters from negative 0.5 to 1.5. The x-axis shows dates from 15-Sep-2003 to 21-Sep-2003. A solid red line for observations and a dashed black line for predictions overlap closely, with K G E 0.80 and N S E 0.97.

At the top center is a bar chart icon labeled Data distribution features.

Panel c, at the top right, contains four satellite photos of different coastal morphologies labeled with north arrows and scale bars, representing Physical conditions such as bathymetry and morphology.

Panel b, in the center, is titled Transfer learning. It contains a map showing three stations: T 1 on an exposed coast, and T 2 and T 3 behind a barrier. Arrows point from T 1 to T 2 and T 3. Below the map is a line graph titled Transfer from T 1 to T 2. The y-axis is Water level in meters from 0 to 3. The x-axis shows dates from 06-Oct-2016 to 12-Oct-2016. A dashed red line for observations sits below a solid black line for predictions, showing degraded performance with Average K G E 0.36 and Average N S E negative 0.37.

At the bottom left is a Neural Network model icon, which receives arrows from panel a and points toward the transfer learning process in panel b.

Navigate back to Figure 2..

### Figure 3. Long description

Panel a is a line graph titled 40 days window, 3 day time step h. The Y-axis represents Water level in meters, ranging from negative 0.5 to 2.0. The X-axis spans from September 1, 2003, to October 8, 2003. Three lines are plotted: a blue line for Actual water level, a green line for N T R, and an orange line for Tide prediction. A vertical orange shaded region around September 18 highlights a significant spike where the actual water level reaches nearly 2.0 meters, driven by a sharp increase in the N T R green line while the tide prediction remains low.

Panel b is a geographical map with Latitude on the Y-axis and Longitude on the X-axis. The map shows a coastal region partitioned into polygonal clusters. Red crosses labeled i sub 1 through i sub 21 mark tide gauge locations. The clusters are concentrated along a central waterway. In the bottom-right of panel b, an inset titled 8-node connectivity shows a central node i with eight arrows pointing outward to numbered nodes 1 through 8 in a compass-like arrangement, representing the spatial information exchange used in the graph convolutional network.

Navigate back to Figure 3..
