# Supplementary material for: Toward transferable models for efficient spatiotemporal flood prediction across coastal-estuarine systems
Source: Camb Prism Coast Futur. 2026 Jun 4;4:e13. doi: 10.1017/cft.2026.10037 (PMC13276722; doi:10.1017/cft.2026.10037)
Supplement: Peer review history

# Reviewer report: Review: Toward transferable models for efficient spatiotemporal flood prediction across coastal-estuarine systems — R0/PR2

**DOI:** 10.1017/cft.2026.10037.pr2

Towards Transferable Models for Efficient Spatiotemporal Flood Prediction Across Coastal Estuarine Systems

This perspective article examines the use of transfer learning (TL) within deep learning (DL) frameworks for predicting extreme water levels (EWLs) and spatiotemporal flood dynamics across coastal-estuarine systems. The authors frame the central problem as one of transferability: how can pretrained DL models be reliably applied from data-rich coastal domains to data-limited or unseen locations?

The manuscript identifies two principal challenges-inaccurate pattern recognition and lagged/hysteresis effects in the spatiotemporal propagation of extreme water levels and traces these to issues of data distribution mismatch, overfitting, and hyperparameter sensitivity. The authors then address what they describe as common misconceptions in current DL flood modeling: training exclusively on extreme events, relying solely on goodness-of-fit metrics, and conflating data-driven pattern detection with physical understanding. In response, they propose a two-step strategy centered on (i) prioritizing storm-relevant signals in feature selection and model architecture (attention mechanisms), and (ii) embedding spatial propagation of EWLs using graph convolutional networks that mimic the physical connectivity of coastal-estuarine hydrodynamics.

Section 5 broadens the discussion to future directions, distinguishing among physics-informed neural networks, differentiable modeling, and physics-guided ML, and advocating for a unified framework that couples neural operators with differentiable hydrologic models to improve boundary conditions and enable real-time ensemble forecasting. The paper concludes with a call for foundation models and formal guidelines for developing robust, interpretable, and generalizable DL models for flood prediction at regional to global scales.

Recommendation: Minor Revisions. Overall, I find the manuscript well conceived and timely and addresses a genuine gap in the ML based flood prediction literature. However, several areas require clarification, additional nuance, or expanded discussion before publication, as detailed below.

Major comments:

• The manuscript focuses on water level prediction but gives limited attention to the full complexity of compound flooding in coastal-estuarine environments. Coastal systems are shaped by simultaneous interactions among storm surge, tidal forcing, riverine discharge, wave setup, rainfall-runoff, and morpho dynamic feedback. The paper mentions some of these drivers (tides, surge, river flow in line 167) but does not adequately discuss how TL frameworks can account for multi-process coupling, nonlinear interactions among drivers. For a journal that emphasizes the complexity of coastal systems, this is a notable gap. Consider expanding Section 2 or Section 4 to address how TL and physics-guided DL architectures can explicitly represent compound flood dynamics, including joint probability of co-occurring drivers, rather than treating water level as a univariate prediction problem.

• The claim that GCN-based spatial propagation is “more consistent with expected coastal-estuarine hydrodynamics” (lines 203–206) is stated as a conceptual argument without empirical comparison against encoder-decoder architectures on shared benchmark domains. Similarly, the discussion of attention mechanisms emphasizing EWL periods (Section 4.1) draws primarily on the authors’ own prior work. A perspective article need not include new experiments, but it should more rigorously synthesize the existing evidence base. I encourage the authors to either cite published benchmarks across multiple TL approaches or, where evidence is absent, explicitly identify these as open research questions.

• I think the distinction among PINNs, differentiable modeling, and physics-guided ML is critically important but is compressed into a single paragraph (lines 227–239). The description of PINNs, focuses narrowly on loss-function residuals without acknowledging their well-documented limitations for complex, multi-scale, and turbulent systems issues directly relevant to coastal dynamics. The statement that DM “is particularly promising because it is more efficient” (line 238) oversimplifies: DM can be more efficient than PINNs for certain forward modeling tasks, but its advantages depend on the specific application, the availability of process equations, and the computational cost of backpropagation through coupled physical modules. It would strengthen the manuscript if the authors can include a more balanced comparison, acknowledge the limitations of each approach in the context of coastal flood prediction, and clarify the conditions under which each is most appropriate.

• The impact statement and conclusion reference “equitable access” (line 48) and “emergency preparedness,” (line 28) but the manuscript does not engage meaningfully with social science perspectives on flood risk, community vulnerability, or the governance challenges of deploying ML based prediction systems. For Cambridge Prisms: Coastal Futures, which explicitly prioritizes interdisciplinary work bridging physical, biological, and social processes, this omission weakens the paper’s fit. A brief discussion of how TL-based flood models interface with decision-making processes, whether outputs are interpretable for emergency managers, and what the implications of model uncertainty are for communities with limited adaptive capacity would help. Adding a paragraph addressing the socio-technical dimensions of model deployment would substantially strengthen the perspective.

• The final paragraphs (section 5.2) say foundation models as the aspirational endpoint for flood prediction, drawing an analogy to large pretrained models in NLP and computer vision. However, the discussion benefits from critical examination of the fundamental differences between language/vision data and geophysical data: coastal systems exhibit extreme spatial heterogeneity, non-stationarity due to climate change and anthropogenic modification, and a relative scarcity of labeled extreme-event data. This section could benefit from addressing the practical barriers to developing hydrologic/hydrodynamic foundation models including data curation at global scales, domain-specific pre-training strategies, and the risk of encoding biases from data-rich regions into models applied to data-sparse areas.

Minor comments:

• line 66: The term “zero-shot application” is introduced without definition. While familiar in the ML community, I wonder coastal science readers may not know whether this refers to application without any local training data, without fine-tuning, or without site-specific calibration. A brief clarification would improve accessibility.

• The illustrative calculation (lines 119 -121) assumes two extreme events per five years lasting 2 to 4 days each over 40 years. This is a useful pedagogical example, but the parameters are somewhat arbitrary and may not generalize well. Extreme event frequency varies enormously by region, and the definition of “extreme” itself (e.g., return period threshold, percentile exceedance) is not specified. Consider either grounding the example in specific observational datasets or explicitly noting the assumptions and their limitations.

• It looks like Shen et al. (2023a) and (2023b) may refer to the same paper, please check if this is a duplication

• In Figure 3b, the inset of 8-node connectivity would benefit from a brief annotation or a more explicit reference in the caption explaining its role in the GCN framework.

• Given that the paper advocates transferable models across diverse coastal systems, a brief discussion of data availability including global tide gauge networks (e.g., GESLA), reanalysis products, and open-source DL toolkits would strengthen the practical relevance. The Data Availability Statement notes that no new data were created, though correct, but a forward-looking discussion of data infrastructure needs would align well with the perspective format.

• The manuscript does not address how sea level rise and long-term non-stationarity in coastal conditions affect the assumptions underlying TL. If source and target domains differ not only in morphology but also in baseline water levels due to secular trends, the similarity of data distributions assumed by TL may degrade over time. This is a critical consideration, especially given the future-oriented scope of the journal and even a brief mention would be helpful

• Line 252-258: The paper discusses sensitivity fidelity briefly in the context of neural operators but does not address uncertainty quantification more broadly. DL models for flood prediction should ideally produce probabilistic outputs or confidence intervals, particularly for emergency decision-making. The authors should note this as an important research direction alongside the other challenges discussed.

# Reviewer report: Review: Toward transferable models for efficient spatiotemporal flood prediction across coastal-estuarine systems — R0/PR3

**DOI:** 10.1017/cft.2026.10037.pr3

This Perspective argues that transfer learning and deep learning can improve efficient spatiotemporal flood prediction across coastal-estuarine systems, especially where data are sparse and physics-based models are expensive to deploy. The manuscript identifies key barriers to transferable flood prediction, including distribution shift, overfitting, hyperparameter dependence, lagged peak timing, hysteresis, poor evaluation metrics, and weak physical interpretability. The topic is timely, interesting, and of great importance. However, I would like to recommend major revision as there are some major issues that need to be addressed.

1-The manuscript moves between transfer learning, GCNs, neural operators, differentiable hydrology, PINNs, and foundation models. The authors may state the most important message and reorganize the manuscript around that argument.

2-Point-gauge prediction, spatial water-level propagation, compound flooding, hydrologic boundary prediction, and neural-operator surrogates are treated together. The authors may either narrow the scope or provide a structured taxonomy separating these modeling tasks.

3-Statements about DL models being comparable to physics-based models and transferable to data-limited regions are too general. The authors should specify the conditions, prediction targets, spatial scales, and evidence supporting these claims.

4-Although written as a Perspective, the manuscript makes broad claims about “current DL approaches” and “common misconceptions.” The authors may explain how studies were selected or add a concise table summarizing representative work.

5-The manuscript implies that attention mechanisms or GCN connectivity can provide physical awareness. The authors should clarify that these tools require physically meaningful constraints, edge definitions, forcing variables, or conservation checks to be hydrodynamically credible.

6-The claim that GCNs are more physically meaningful than U-Net-like interpolation is not fully justified.

7-Operational flood prediction requires uncertainty estimates. The authors should add a section on probabilistic forecasting, ensemble methods, calibration, and decision-relevant uncertainty in fluvial, coastal or pluvial floods such as data assimilation and recently developed AI-based data assimilation methods that are relevant to the topic.

8-The manuscript mentions coastal, pluvial, and fluvial drivers but mainly focuses on water-level propagation from gauges. The authors should discuss how transfer learning handles interacting surge, tide, rainfall, runoff, waves, and backwater effects.

# Editor report: Recommendation: Toward transferable models for efficient spatiotemporal flood prediction across coastal-estuarine systems — R0/PR4

**DOI:** 10.1017/cft.2026.10037.pr4

**Affiliations:** School of Geosciences, University of South Florida, United States

Dear Authors,

Thank you for submitting your manuscript to the journal. This is an interesting and timely work that fits well within the journal’s scope, and the paper itself is in very good shape.

Both reviewers raised similar comments, pointing to comparable issues from different perspectives. I agree with the feedback from both, particularly regarding the inclusion of a discussion of compound flooding and the importance of making the vocabulary, methodology, and overall context more accessible to coastal scientists who may not be familiar with machine learning. However, I agree that the compound flooding discussion cannot be extensive, in order to comply with the length limitation (word count). I also agree that the manuscript would benefit from some restructuring.

One reviewer formally recommended “Major Revision”; however, I believe the changes requested are relatively minor. This is somewhat subjective, but the revisions do not, in my view, affect the core of the paper.

I congratulate the authors on a well-executed study that will make a valuable contribution to the literature on flood modeling using machine learning.

I look forward to receiving the revised manuscript.

Best regards,

Alex Enriquez

Handling Editor

# Editor report: Decision: Toward transferable models for efficient spatiotemporal flood prediction across coastal-estuarine systems — R0/PR5

**DOI:** 10.1017/cft.2026.10037.pr5

**Affiliations:** Faculty of Engineering, UBC Institute for the Oceans and Fisheries, Canada

No accompanying comment.

# Editor report: Recommendation: Toward transferable models for efficient spatiotemporal flood prediction across coastal-estuarine systems — R1/PR7

**DOI:** 10.1017/cft.2026.10037.pr7

**Affiliations:** School of Geosciences, University of South Florida, United States

Overall, the authors have addressed the reviewers' suggestions and resolved all concerns. Some modifications were not as extensive as requested, due to word count constraints and to maintain the paper’s focus — I agree with the authors on this. There are other comments that were justifiably not implemented. I would suggest re-sending the paper to the reviewers to confirm that the modifications satisfy their concerns. More details follow below.

One issue raised by both reviewers was the need for further discussion of compound flooding. The authors addressed compound flooding in different points throughout the paper. Although a more in-depth discussion was requested, the authors have included the relevant information without exceeding the word count, and I find this appropriate. The paper’s central focus is on ML transferability rather than compound flooding; a more extended discussion on compound flooding could detract from that focus.

Some comments from Reviewer #2 were addresed but not implemented, including the reorganization of the paper and the inclusion of different model configurations (point-gauge, spatial water level, compound floods, etc.). The authors explicitly explain their reasons for not following these suggestions. I would return the manuscript to the reviewer to asses whether the revisions are satisfactory.

# Editor report: Decision: Toward transferable models for efficient spatiotemporal flood prediction across coastal-estuarine systems — R1/PR8

**DOI:** 10.1017/cft.2026.10037.pr8

**Affiliations:** Geography, Cambridge University, United Kingdom of Great Britain and Northern Ireland

No accompanying comment.
